# Blood-based non-invasive tests show comparable accuracy in MetALD and MASLD for MRE-defined advanced fibrosis

**DOI:** 10.1186/s12876-026-04839-w

**Published:** 2026-04-18

**Authors:** Akash Roy, Surabhi Jajodia, Shardhya Chakraborty, Shruti Keyal, Awanish Tewari, Nikhil Sonthalia, Sourish Roy, Rajat Khandelwal, Anand Kulkarni, Uday Chand Ghoshal, Rajender Reddy, Mahesh Kumar Goenka

**Affiliations:** 1 Institute of Gastrosciences and Liver Transplant, Apollo Multispeciality Hospitals, Kolkata, West Bengal 700054 India; 2 Department of Radiodiagnosis and Imaging, Apollo Multispeciality Hospitals, Kolkata, 700054 India; 3 Department of Hepatology, Asian Institute of Gastroenterology, Hyderabad, 500032 India; 4 Division of Gastroenterology and Hepatology, University of Pennsylvania, 2 Dulles, Liver Transplant Office 3400 Spruce Street, Philadelphia, PA 19104 USA

**Keywords:** MetALD, FIB-4, NAFLD fibrosis score, Magnetic resonance elastography (MRE), Advanced fibrosis, Diagnostic accuracy

## Abstract

**Background:**

Non-invasive tests (NITs) triage individuals with metabolic dysfunction-associated steatotic liver disease (MASLD) for advanced fibrosis (AF). However, evidence for performance in metabolic dysfunction and alcohol-associated liver disease (MetALD) remains limited.

**Aim:**

To determine how commonly used NITs perform in MetALD and MASLD using magnetic resonance elastography (MRE) as the reference.

**Methods:**

Adults (≥ 18 years) with MASLD /MetALD with cardiometabolic risk factors (CMRFs) and MRI proton density fat fraction-defined steatosis were included. Fibrosis-4 (FIB-4), AST platelet ratio index (APRIF), and NAFLD fibrosis score (NFS) were estimated. Contemporaneous MRE served as the comparator with a cut-off for AF of 3.53 kPa and sensitivity analysis at 3.6 kPa.

**Results:**

Among 1010 individuals [44 (35-54 years), 35.2% females], 842(83.3%) had MASLD and 168(16.6%) had MetALD. CMRF [diabetes,36.6% vs.30.9%, *p* = 0.15], hypertension (38.2% vs. 34.5% *p* = 0.36), dyslipidaemia (31.5% vs. 33.9%, *p* = 0.55), and burden of AF (15.4% vs. 18.4%, *p* = 0.33) were similar. APRI was suboptimal [AUC 0.63 (0.60–0.66)]. In MASLD, the AUC for FIB-4 and NFS were 0.77 (0.72–0.82) and 0.80 (0.76–0.85), respectively. For MetALD, the AUC of FIB-4 and NFS were 0.78 (0.68–0.87) and 0.81 (0.70–0.89), respectively. FIB-4 (AUC 0.770 vs. 0.782, *p* = 0.99) and NFS (AUC 0.804 vs. 0.80, *p* = 0.94) had similar performance. Sensitivity analysis at 3.6 kPa threshold yielded similar results.

**Conclusion:**

In a liver clinic cohort, MASLD and MetALD showed similar CMRF and AF burden. FIB-4 and NFS, but not APRI, show clinically useful and similar performance for AF, supporting inexpensive standard NIT-based triage in MetALD.

**Graphical abstract:**

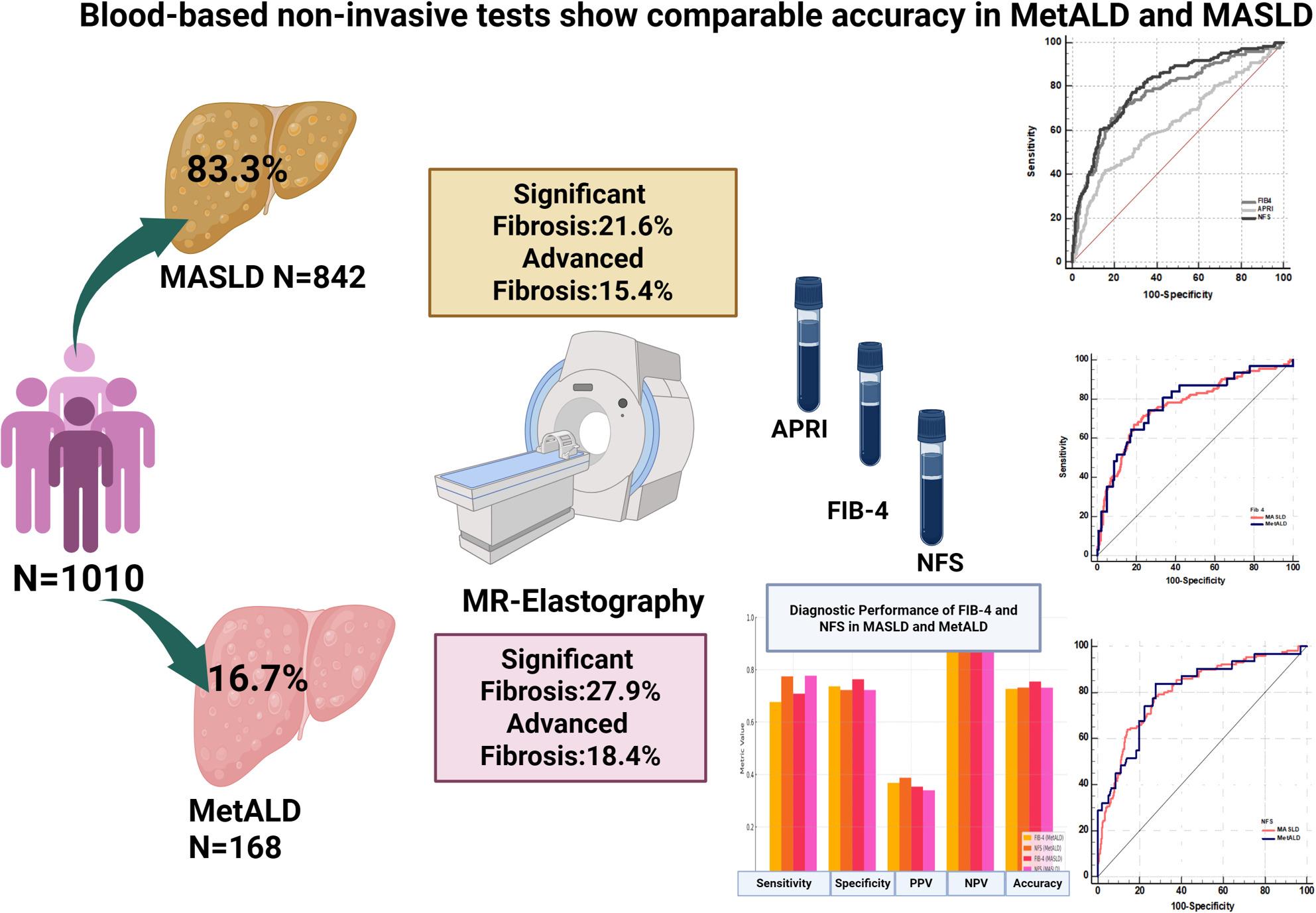

## Introduction

Recently, the nomenclature of steatotic liver disease (SLD) has been adopted globally and marks a paradigm shift in the understanding of fatty liver disease [1, 2]. The new nomenclature essentially focuses on the presence of cardiometabolic risk factors (CMRF) and allows for the incorporation of specific thresholds for alcohol intake [2]. Accordingly, SLD is stratified as metabolic dysfunction-associated steatotic liver disease (MASLD) and Alcohol-associated liver disease at the two extremes and an intermediate overlap phenotype of MetALD [2, 3].

SLD has fast emerged as a global health concern with a high disease burden, with recent estimates suggesting that one in three adults has MASLD [4, 5]. Given the high overall burden, identifying those at greatest risk has been a key strategy in stratifying MASLD [6]. Accordingly, the use of simple, non-invasive tests, such as the Fibrosis-4 (FIB-4), to identify those at risk of advanced fibrosis has been endorsed by most society guidelines [3, 7]. Robust metrics on the performance of such stratification pathways are available from the era when the term non-alcoholic fatty liver disease (NAFLD) was commonly used [8]. However, in the context of the new nomenclature, the performance of NITs, especially in the MetALD group, has limited literature. A recent report from a Korean health check-up cohort found comparable performance of the FIB-4 and NAFLD Fibrosis Score (NFS) in predicting advanced fibrosis in MetALD and MASLD [9]. However, contradictory observations from a Japanese health check-up cohort noted poor performance of FIB-4 compared with vibration-controlled elastography (VCTE)- based liver stiffness measurement [10].

These estimates from health check-up cohorts arise from a low-prevalence screening setting, which differs from liver clinics, where pre-test probability is substantially higher [11]. Hence, whether NITs are equivalent in MASLD and MetALD when applied in clinical settings remains uncertain. Additionally, the prior health-check cohorts defined steatosis by ultrasound, which is prone to operator variability and reduced sensitivity. Modalities such as magnetic resonance imaging proton density fat fraction (MRI-PDFF), have been shown to be more accurate [12].

In this context, we hypothesized that NITs may exhibit similar operating performance for advanced fibrosis in MASLD and MetALD within a liver clinic cohort and evaluated FIB-4, NFS, and AST/Platelet Ratio index (APRI), and, thus, endeavoured to validate it through contemporaneous MRE and MRI-PDFF, a modality that is known to have low variability.

## Methods

### Study design

This was a retrospective analysis of a prospectively maintained database for individuals with SLD in a dedicated liver clinic at a tertiary care centre between January 2024 and July 2025, and followed the Standards for Reporting of Diagnostic Accuracy (STARD) guidelines (Supplementary Table 1).

### Inclusion and exclusion criteria for participants

Adults aged 18 years or older attending the liver clinic were eligible if they had evidence of hepatic steatosis confirmed by MRI-PDFF ≥ 6.4%, met MASLD or MetALD criteria, and underwent magnetic resonance elastography (MRE) within 30 days of the laboratory tests used to compute values for non-invasive tests.

**Fig. 1:**
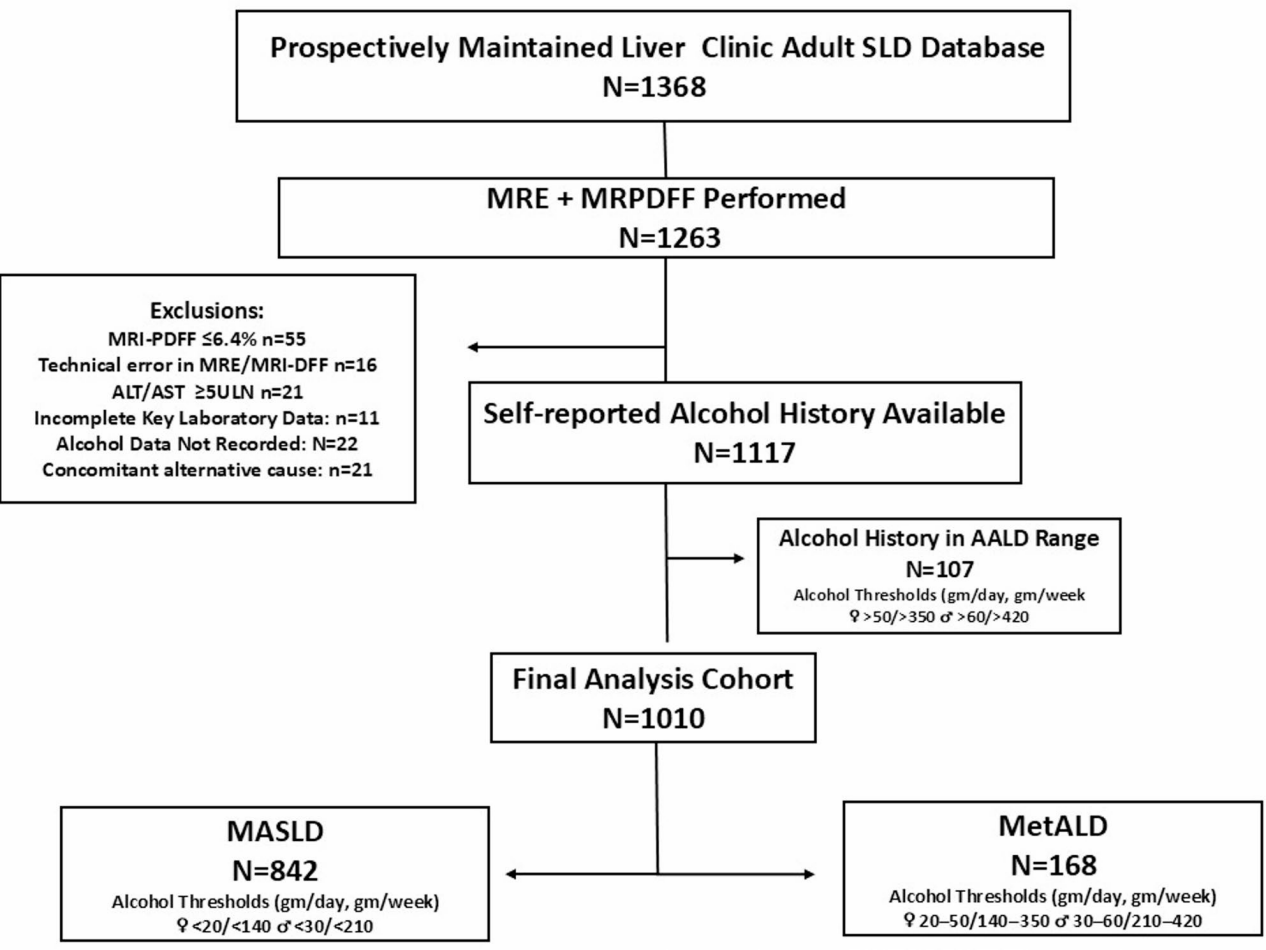
Flow chart of participants

Individuals with non-diagnostic MRI-PDFF/MRE studies (technical failure, motion artefacts, excessive iron, or MRI contraindications) and incomplete key laboratory data or alcohol history were excluded. Additionally, the presence or history of an alternative liver disease aetiology (hepatitis B, hepatitis C, autoimmune hepatitis, drugs causing steatosis, ) or elevated aminotransferase which may interfere with MRE interpretation, (ALT > 5 times ULN), presence of cholestasis or biliary obstruction, clinical evidence of right sided heart failure, pregnancy, and active hepatic or extrahepatic malignancy were excluded. A flow diagram illustrating the inclusion and exclusion criteria is shown in Fig. 1.

### Index tests and reference standards

We evaluated three routinely available, blood-based NITs, namely FIB-4, APRI, and the NFS score calculated from standard clinical and laboratory parameters obtained contemporaneously with MRE. FIB-4 integrates age, aminotransferases, and platelet count; APRI incorporates AST (normalized to the laboratory upper limit of normal) and platelet count; and NFS combines demographic, metabolic and biochemical variables. These NITs were selected because they are inexpensive, widely implemented in fibrosis triage pathways, and do not require proprietary assays. Since age inflates the FIB-4, we prespecified age-adjusted operating points: for patients aged 65 years or older, we used a cutoff of < 2 for ruling out advanced fibrosis. The reference standard test was MRE-derived liver stiffness. Advanced fibrosis (AF) was defined a priori as MRE ≥ 3.53 kPa, with a threshold of ≥ 3.60 kPa tested in sensitivity analyses [13]. Significant fibrosis (SF) was defined as MRE ≥ 3.14 kPa [13]. MRE stiffness values were extracted from finalized clinical reports. Technical acquisition parameters and quality-control criteria were followed according to local protocol, as outlined later.

### MASLD CMRF and alcohol usage definition

Cardiometabolic criteria for defining MASLD were defined as having at least one of the 5 criteria from below.

- BMI ≥ 23 kg/m^2^ (as per Asian Cut-offs)(Only BMI criteria was considered).
- Fasting blood glucose > 100 mg/dl or HBA1c > 5.7 or treatment for diabetes mellitus.
- Blood pressure > 135/80 mm Hg or treatment for hypertension.
- Plasma triglycerides > 150 or treatment with lipid-lowering drugs.
- Plasma high-density lipoprotein (HDL) < 40 mg /dl for males or < 50 mg/dl for females, or treatment with lipid-lowering drugs.

Self-reported AC history was recorded and quantified in grams/day or per week, as applicable. Thresholds for AC for defining the intermediate group MetALD were 140–350 gm/week (or 20–50 gm/day) for females and 210–420 gm/week (or 30-60gm/day) for males. Those below these thresholds were considered to have MASLD, and those above, ALD as defined by the current nomenclature systems for steatotic liver disease classification [2].

#### MR techniques

Magnetic resonance (MR) images were obtained using a 3.0-T device (Philips 3.0 Tesla with Spin–Echo Echo–Planar Imaging protocol). Once the patient was on the examination table, a pneumatic driver was placed over the upper abdomen to generate mechanical acoustic waves to the liver. The driver’s operating frequency was set to approximately 60 Hz. MR elastography SE-EPI sequence was obtained using a body coil and the following parameters: TR, TE 1066,58 msec; flip angle 90 degrees; field of view 40-40-8.7 cm; matrix 256 × 64; thickness 10 mm. Once the images were acquired, the data were automatically post-processed to generate a stiffness map. The shear stiffness was measured in kPa as a mean value from the eight representative regions of interest (ROI) across eight separate slices by trained radiologists blinded to clinical data. MRE scans with confidence maps showing > 50% of the ROI in low-confidence regions were excluded as non-diagnostic. MRI-PDFF was also obtained using the 3.0 Tesla Phillips device, and Dixon Quant software was used. MRI-PDFF values greater than 6.4% indicated steatosis according to established cut-offs [14].

### Sample size

In an enriched setting, such as a liver clinic, the prevalence of advanced fibrosis ranges from 18% to 27% [15]. Recent guidelines consistently demonstrate that FIB-4 and NFS have moderate discrimination for AF, with area under the receiver operating characteristic curves (AUROC) ranging from 0.75 to 0.78 [3]. Hence, for the primary accuracy objective, we planned the sample size to estimate the AUROC of the index tests for MRE-defined advanced fibrosis with a two-sided 95% confidence interval (CI) half-width ≤ 0.05. Using the Hanley–McNeil variance for AUROC and assuming a conservative expected AUROC of 0.78 and a clinic prevalence of advanced fibrosis of 18%, the required total sample is at least 700 to achieve the targeted precision.

#### Statistical analysis

The data were presented as the mean ± SD or median (IQR) for quantitative variables, depending on the data’s normality. Qualitative variables were expressed as n (%). The Student’s t-test was used for continuous variables, whereas the Chi-square or Fisher’s exact test was used for categorical variables. We analyzed the overall cohort and by etiology (MASLD, MetALD). The primary endpoint was advanced fibrosis (AF) by MRE ≥ 3.53 kPa. We computed FIB-4, NFS, and APRI using standard formulae and prespecified cut-offs: FIB-4 low < 1.30 if age < 65 or < 2.00 if ≥ 65, high ≥ 2.67; NFS low < − 1.455, high ≥ 0.676; APRI low < 0.50, high ≥ 1.50. Discrimination was quantified by area under receiver operating curves (AUROC) with 95% CIs via 1,000-sample bootstrap, and within-patient AUROC differences (FIB-4 vs. NFS) by DeLong’s test. At the prespecified cut-offs, we reported sensitivity, specificity, positive predictive value (PPV), and negative predictive value (NPV).

#### Sensitivity analysis

As an alternative advanced fibrosis threshold of > 3.60 kPa has also been suggested for MRE, we conducted an exploratory sensitivity analysis using this threshold [16].

#### Missing data

Among the variables in the MASLD definition, HDL had the lowest missingness rate (3.6%). Given the low rate of missing data and minimal interference with the primary endpoint, no imputation was performed. Two-sided α = 0.05 defined significance.

All statistical analyses and figures were prepared using IBM SPSS Statistics v26.0 (IBM Corp., Armonk, NY, USA), MedCalc v20.0 (MedCalc Software Ltd., Mariakerke, Belgium), GraphPad Prism (GraphPad Software, San Diego, CA, USA), and Python (Matplotlib).

#### Ethical statement

Prior ethical committee approval obtained from the Institutional Ethical Committee of Apollo Multiscpeciality Hospitals, vide number IEC/BR/2024/03/06, and the study adhered to the Declaration of Helsinki, with consent to participate being obtained.

## Results

### Baseline characteristics

Of the 1368 individuals aged 18 years and older, after exclusions, 1010 were included in the study cohort. 842 (83.3%) had MASLD, whereas 168 (16.6%) were categorized as MetALD. The baseline characteristics of the entire cohort and stratification as MASLD and MetALD is shown in Table 1. Individuals with MetALD were younger [40(34–51 vs. 45 (35–55) years] and overwhelmingly male (94.6% vs. 58.7%). CMRFs were similar between the two groups. Each additional CMRF was associated with higher odds of MRE-defined advanced fibrosis. In the overall cohort, the odds ratio (OR) was 1.53 (95% CI 1.32–1.76; *p* < 0.001). In MASLD, the OR per additional CMRF was 1.51 (95% CI 1.29–1.77; *p* < 0.001), and in MetALD, the corresponding OR, was 1.63 (95% CI 1.16–2.28; *p* = 0.004). Individuals with MetALD had higher ALT [ 60.0 (36.0–89.0) vs. 50.0 (30.0–68.0) IU/L]. Both the presence of MRE-defined advanced fibrosis and significant fibrosis between MASLD and MetALD were similar (15.4% vs. 18.4%, *p* = 0.33; & 21.6% vs. 27.9%, *p* = 0.07, respectively).

**Table 1:** Baseline characteristics of the overall cohort and comparison between individuals with MASLD and MetALD

Variable	Overall (*n* = 1010)	MASLD (*n* = 842)	MetALD (*n* = 168)	*p*-value
Age (years)	44.0 (35.0–54.0)	45.0 (35.0–55.0)	40.0 (34.0–51.0)	< 0.01
Gender (Male), n (%)	653/1010 (64.7)	494/842 (58.7)	159/168 (94.6)	< 0.001
Height (cm)	165.0 (156.0-170.0)	163.0 (155.0-170.0)	169.0 (164.8–173.0)	< 0.001
Weight (kg)	74.0 (65.6–83.0)	73.0 (65.0–82.0)	79.7 (71.0-88.5)	< 0.001
BMI (kg/m²)	27.3 (24.9–30.4)	27.2 (24.8–30.5)	27.6 (25.3–30.4)	0.37
Hypertension, n (%)	380/1010 (37.6)	322/842 (38.2)	58/168 (34.5)	0.41
Diabetes mellitus, n (%)	361/1010 (35.7)	309/842 (36.7)	52/168 (31.0)	0.18
Dyslipidemia, n (%)	323/1010 (32.0)	266/842 (31.6)	57/168 (33.9)	0.61
Thyroid disease, n (%)	169/1010 (16.7)	149/842 (17.7)	20/168 (11.9)	0.08
CAD, n (%)	40/1010 (4.0)	34/842 (4.0)	6/168 (3.6)	0.94
CKD, n (%)	14/1010 (1.4)	12/842 (1.4)	2/168 (1.2)	1.00
COPD, n (%)	31/1010 (3.1)	24/842 (2.9)	7/168 (4.2)	0.51
Smoking, n (%)	212/1010 (21.0)	125/842 (14.8)	87/168 (51.8)	< 0.001
Hemoglobin (g/dL)	12.5 (11.9–14.1)	12.3 (11.7–14.0)	13.9 (12.0-14.8)	< 0.001
Platelets (×10³/µL)	201.0 (161.0-225.0)	201.0 (161.2–225.0)	200.2 (159.8-215.2)	0.39
ALT (U/L)	51.0 (31.0–74.0)	50.0 (30.0–68.0)	60.0 (36.0–89.0)	< 0.001
AST (U/L)	46.0 (30.0–54.0)	46.0 (29.0–54.0)	46.0 (31.8–58.0)	0.24
ALP (U/L)	101.0 (80.0-103.0)	101.0 (81.0-104.0)	100.3 (78.8–101.0)	0.05
Albumin (g/dL)	4.2 (4.1–4.6)	4.2 (4.1–4.5)	4.3 (4.1–4.6)	0.07
Creatinine (mg/dL)	0.9 (0.8–0.9)	0.9 (0.8-1.0)	0.9 (0.8–0.9)	0.47
HbA1c (%)	6.2 (5.6–7.2)	6.3 (5.6–7.2)	5.9 (5.4–7.1)	0.15
Fasting glucose (mg/dL)	102.0 (90.5–128.0)	103.0 (91–129)	97.0 (89-126.5)	0.17
Triglycerides (mg/dL)	140 (108–167)	140 (108–167)	141 (108-170.2)	0.26
HDL cholesterol (mg/dL)	42.15(11.1)	42.1(11.2)	41.4 (11.7)	0.70
FIB-4	1.3 (0.9–1.9)	1.3 (0.9-2.0)	1.2 (0.9–1.8)	0.20
APRI	0.6 (0.4–0.8)	0.6 (0.4–0.8)	0.6 (0.4–0.8)	0.12
NFS	-1.7 (-2.6–0.5)	-1.6 (-2.5–0.4)	-2.0 (-2.8–0.9)	0.03
MRE stiffness (kPa)	2.3 (2.2-3.0)	2.3 (2.2–2.9)	2.3 (2.1–3.2)	0.93
PDFF (%)	13.7 ± 4.8	13.7 ± 4.7	13.5 ± 4.9	0.53
Significant fibrosis, n (%)(MRE ≥ 3.14)	229(22.7)	182(21.6)	47 (27.9)	0.09
Advanced fibrosis, n (%)(MRE ≥ 3.53)	161(15.9)	130(15.4)	31 (18.4)	0.39

*HDL was available for 96.4% of patients; missing values (3.6%) were not imputed and do not affect primary analyses

*ALT* Alanine transaminase, *AST* Aspartate transaminase, *ALP* Alkaline phosphatase, *CAD* Coronary artery disease, *CKD* Chronic kidney disease, *BMI* Body mass index, *MRE* Magnetic resonance elastography, *MRI-PDFF* Magnetic resonance imaging proton density fat fraction, *HDL* High density lipoprotein, *APRI* AST to Platelet Ratio Index, *NFS* NAFLD Fibrosis Score, *FIB-4* Fibrosis 4

### Diagnostic accuracy at primary threshold

Overall, for advanced fibrosis, the AUROC for FIB-4, NFS, and APRI were 0.77 (0.72–0.81), 0.80 (0.76–0.84), and 0.63 (0.57–0.68), respectively. In individuals with MASLD, the AUROC values for FIB-4 and NFS were 0.77 (0.72–0.82) and 0.80 (0.76–0.85), respectively. For MetALD, the AUROC for FIB-4 and NFS was 0.78 (0.68–0.87) and 0.81 (0.70–0.89), respectively. Across the entire cohort and among MASLD and MetALD, both FIB-4 and NFS had negative predictive values of above 90%. Table 2 and Supplementary Fig. 1 show the diagnostic performance of the tests in the cohort reflecting the similar performance of FIB-4 and NFS and suboptimal performance of APRI.

**Table 2:** Performance of non-invasive tests for advanced fibrosis defined by magnetic resonance elastography cut-off of 3.53 kPa

Cohort	Test	AUC (95% CI)	Sensitivity*	Specificity*	NPV*	Sensitivity**	Specificity**	PPV**
Overall	FIB-4	0.77 (0.72–0.81)	78.88	56.77	93.41	39.75	91.40	46.72
	APRI	0.63 (0.57–0.68)	69.57	43.11	88.19	16.15	94.58	36.11
	NFS	0.80 (0.76–0.84)	83.85	62.03	95.29	31.68	94.69	53.12
MASLD	FIB-4	0.77 (0.72–0.82)	79.23	55.76	93.63	40.77	90.87	44.92
	APRI	0.64 (0.59–0.69)	70.00	44.10	88.95	16.92	95.22	39.29
	NFS	0.80 (0.76–0.85)	85.38	59.92	95.73	31.54	94.09	49.40
MetALD	FIB-4	0.78 (0.68–0.87)	77.42	62.04	92.39	35.48	94.16	57.89
	APRI	0.58 (0.46–0.70)	67.74	37.96	83.87	12.90	91.24	25.00
	NFS	0.81 (0.70–0.89)	77.42	72.99	93.46	32.26	97.81	76.92

*AUC* Area Under the Curve, *PPV* Positive Predictive Value, *NPV* Negative Predictive Value, *MASLD* Metabolic dysfunction-Associated Steatotic Liver Disease, *MetALD* Metabolic dysfunction and Alcohol-associated Liver Disease, *APRI* AST to Platelet Ratio Index, *NFS* NAFLD Fibrosis Score; *Lower cut off; ** higher cut off; FIB-4, Fibrosis 4;FIB-4 lower cutoff < 1.30 (or < 2.00 for age ≥ 65), upper cutoff ≥ 2.67; NFS lower cutoff < − 1.455, upper cutoff ≥ 0.676; APRI lower cutoff < 0.50, upper cutoff ≥ 1.50

### Within-etiology and between-aetiology comparisons

**Fig. 2:**
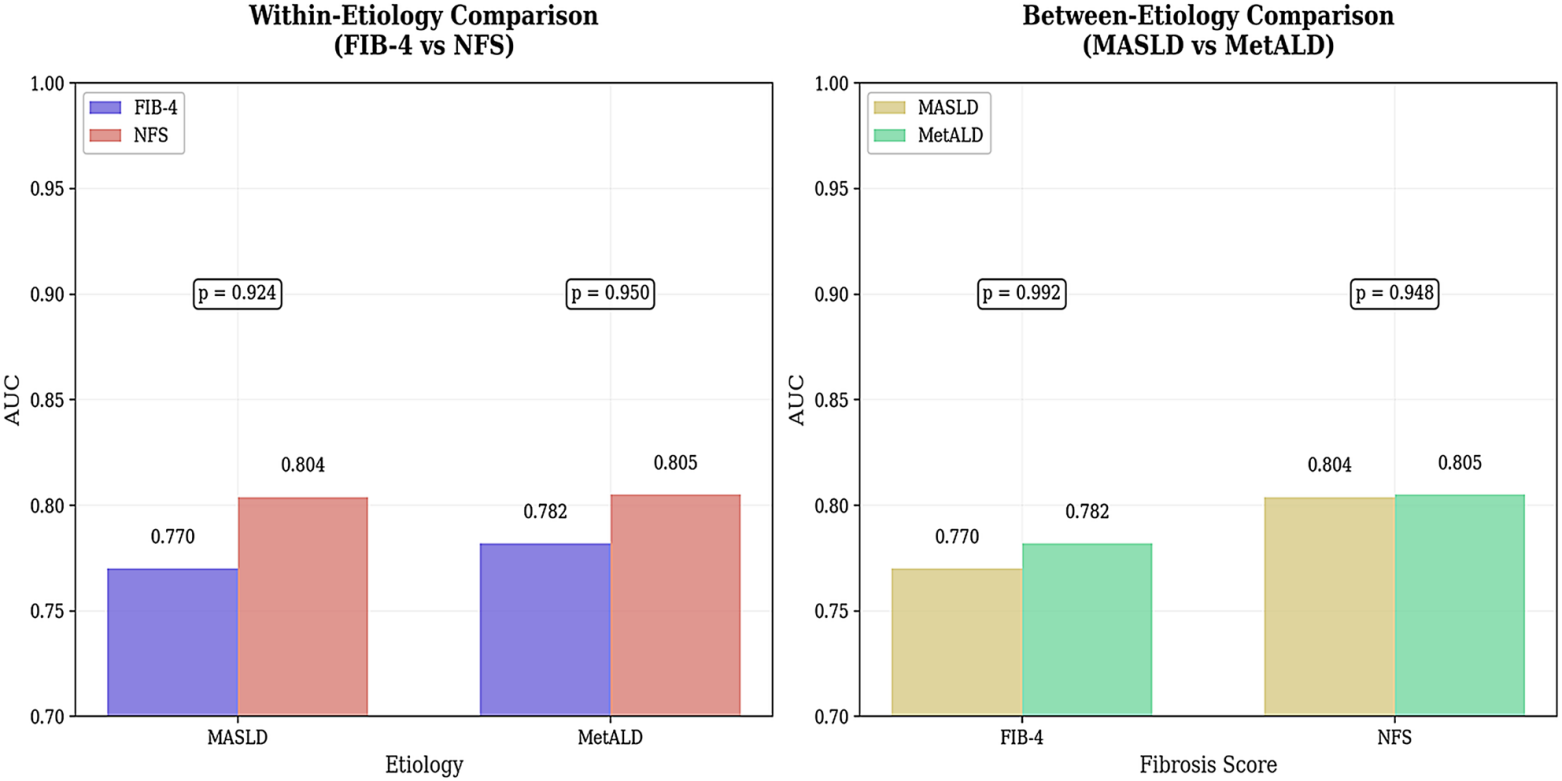
Comparison between FIB-4 and NFS within and between aetiology (MASLD vs. Met ALD)

In individuals with MASLD, NFS slightly outperformed FIB-4 (AUC 0.804 vs. 0.770), but the difference was not statistically significant (*p* = 0.92). A similar pattern was observed in MetALD with NFS, showing marginally better performance than FIB-4 (AUC 0.805 vs. 0.782), but the difference was not statistically significant (*p* = 0.95). FIB-4 had virtually similar performance between MASLD and MetALD (AUC 0.770 vs. 0.782, *p* = 0.992), which was also the case for NFS (AUC 0.804 vs. 0.805, *p* = 0.948) (Fig. 2).

Age-stratified analysis showed that in patients aged < 65 years, the AUROC of FIB-4 was 0.75 for MASLD and 0.79 for MetALD, while the AUROC of NFS was 0.79 for MASLD and 0.80 for MetALD; APRI showed lower discrimination, with AUROCs of 0.63 for MASLD and 0.57 for MetALD. In patients aged ≥ 65 years, the AUROC of FIB-4, NFS, and APRI in MASLD was 0.78, 0.75, and 0.67, respectively. However, the ≥ 65-year MetALD subgroup was very small (*n* = 7), precluding meaningful analysis.

### Sensitivity analysis

Sensitivity analysis using MRE ≥ 3.60 kPa for advanced fibrosis revealed AUROCs of 0.77 (0.73–0.81) for FIB-4, 0.80 (0.76–0.84) for NFS, and 0.63 (0.58–0.68) for APRI, respectively. Similar to a cutoff of 3.53, the NPVs for both FIB-4 and NFS were above 90% regardless of aetiology (MASLD or MetALD) (Table 3).

**Table 3:** Sensitivity analysis for performance of NITs with advanced fibrosis defined as MRE 3.6 kPa

Cohort	Test	AUC (95% CI)	Sensitivity*	Specificity*	NPV*	Sensitivity**	Specificity**	PPV**
Overall	APRI	0.63 (0.58–0.68)	69.18	43.01	88.19	16.35	94.59	36.11
	FIB-4	0.77 (0.73–0.81)	79.25	56.76	93.60	40.25	91.42	46.72
	NFS	0.80 (0.76–0.84)	84.28	62.00	95.47	32.08	94.71	53.12
MASLD	APRI	0.64 (0.58–0.70)	69.53	43.98	88.95	17.19	95.24	39.29
	FIB-4	0.77 (0.72–0.82)	79.69	55.74	93.87	41.41	90.90	44.92
	NFS	0.80 (0.76–0.85)	85.94	59.89	95.96	32.03	94.11	49.40
MetALD	APRI	0.58 (0.46–0.70)	67.74	37.96	83.87	12.90	91.24	25.00
	FIB-4	0.78 (0.68–0.87)	77.42	62.04	92.39	35.48	94.16	57.89
	NFS	0.81 (0.70–0.89)	77.42	72.99	93.46	32.26	97.81	76.92

*AUC* Area Under the Curve, *PPV* Positive Predictive Value, *NPV* Negative Predictive Value, *MASLD* Metabolic dysfunction-Associated Steatotic Liver Disease, *MetALD* Metabolic dysfunction and Alcohol-associated Liver Disease, *APRI* AST to Platelet Ratio Index, *NFS* NAFLD Fibrosis Score; *Lower cut off; ** higher cut off; FIB-4, Fibrosis 4;;FIB-4 lower cutoff < 1.30 (or < 2.00 for age ≥ 65), upper cutoff ≥ 2.67; NFS lower cutoff < − 1.455, upper cutoff ≥ 0.676; APRI lower cutoff < 0.50, upper cutoff ≥ 1.50

## Discussion

In a tertiary care liver clinic cohort enriched for cardiometabolic risk factors, phenotyped using MRI-PDFF for steatosis and MRE for fibrosis, we evaluated the diagnostic performance of commonly used blood-based NITs compared with MRE-defined advanced fibrosis for MASLD and MetALD. The principal findings are threefold. Both FIB-4 and NFS demonstrate acceptable performance, with high negative predictive values for excluding advanced fibrosis across the cohort. APRI underperformed across MASLD and MetALD, reflecting its limitation as a tool for fibrosis stratification. Between MASLD and MetALD, both FIB-4 and NFS showed no significant differences and behaved similarly, thereby highlighting the potential of such simple NITs as reliable risk stratification tools, even for MetALD, within the new SLD classification.

Blood-based NIT-based stratification has been universally recommended in NAFLD care pathways, given the high disease burden and the need to identify those at risk [3, 17]. Across practice guidance documents, FIB-4 has been recommended as the initial risk assessment tool, on account of its excellent negative predictive value with secondary assessment by elastography or other tests (e.g. enhanced liver fibrosis score) when FIB-4 is indeterminate or elevated. This stratification pathway is explicitly designed to function across care settings, with MRE positioned as an appropriate next-step tool in higher-prevalence specialty clinics when uncertainty persists [18, 19].

The performance of FIB-4 for such risk stratification for NAFLD is well established [18]. However, in the context of the revised SLD nomenclature, there is a need to assess the performance of these pathways across the new phenotypes. MASLD is very similar to and has considerable overlap with the previous NALFD nomenclature [20]. However, the incorporation of MetALD as a new subgroup is a key feature of the new nomenclature system, and whether conventional blood-based NITs, proposed for NAFLD/MASLD, work in MetALD remains an open question.

Limited data exist on the applicability of NITs for MetALD. The largest evidence to date comes from a large health check-up cohort in Japan, which used ultrasound-defined steatosis and MRE in 3,638 individuals [9]. The prevalence of MetALD was 5.8%, and 1.5% had advanced hepatic fibrosis. Both MetALD and MASLD displayed similar metabolic profiles and hepatic fibrosis burdens. The diagnostic performance of FIB-4 and NFS showed no noticeable differences in AUROCs [9]. This is a classic low-prevalence cohort of MASLD, especially seen in primary care and health check-up cohorts, but is limited by ultrasound as a mode to define steatosis. Complementing this was the “San Diego Liver Study,” with 617 individuals, of whom 97 (15.7%) had MetALD. FIB-4 ≥ 1.3 demonstrated good performance (AUROC: 0.78, 95% CI: 0.58–0.98, and NPV: 98%), supporting the feasibility of a step-wise approach starting with FIB-4 in MetALD, with a low misclassification rate [21]. The third study emerges from a European cohort of 423 SLD patients, of whom 102 (24%) had MetALD, as classified using histology or controlled attenuation parameter for steatosis and self-reported alcohol intake. The study reinforced the recommendation that the cut-offs for diagnosing advanced fibrosis are applicable to the new MetALD subclass. Additionally, a two-step sequential testing strategy with FIB- 4 and transient elastography led to the identification of a MetALD patient subgroup with a high risk of later hepatic decompensation [22]. Thus, across cohorts with varying pre-test probability and outcome measures for defining steatosis and fibrosis, similar themes emerge. Hence, by unifying the emerging literature with our findings, it may be suggested that a sequential NIT-based clinical pathway, which has already been shown to perform in MASLD, may also be applicable to MetALD, with further validation in larger MetALD cohorts.

The current study was conducted in a specialty liver clinic, which is enriched for higher-risk phenotypes; hence, the pre-test probability of advanced fibrosis is higher than in population screening cohorts. As the NIT utility is prevalence-dependent, demonstrating preserved rule-out performance strengthens its real-world applicability [23]. Despite intrinsic differences in the study cohorts compared with other studies, we find a similar pattern of performance equivalence between FIB-4 and NFS, irrespective of MASLD or MetALD as the aetiology. These findings also support the biological plausibility that simple blood-based NITs (such as FIB-4 and NFS) behave similarly in MASLD and MetALD, because, although alcohol exposure is accounted for, cardiometabolic dysfunction is also central to MetALD. Hence, the scores that primarily capture fibrosis burden rather than aetiology-specific signatures may be expected to have similar outcomes in MASLD and MetALD. A logical next step may be to test sequential and combination NIT pathways in MetALD, analogous to MASLD care algorithms such as MEFIB (MRE and FIB-4), which may help enrich for clinically meaningful fibrosis in liver clinic settings and also assess prognostic implications [24].

The current study has several strengths, including a real-world liver clinic design with a large dataset and a short interval between laboratory indices and MRE, as well as the use of MRI-PDFF for inclusion, which avoids potential ultrasound misclassification. Fat fraction assessment by any modality like MRPDFF and new evolving ultrasound-based fat fractions has been shown to accurately replicate histological steatosis [25]. Additionally, sensitivity analysis was performed at an alternative MRE threshold for advanced fibrosis. However, formal intra- or inter-test reproducibility testing was not performed specifically for this analysis.

However, the study has several limitations, including a retrospective design, albeit based on a carefully maintained prospective database. It should be emphasized that this is a single-centre tertiary hepatology clinic cohort with MRI-PDFF and MRE availability, which results in enrichment for individuals with higher clinical suspicion for significant liver disease and may not translate to non-enriched settings such as primary care. Lack of formal intra- or inter-test reproducibility testing remains another limitation. Alcohol usage was self-reported, which introduces an element of potential misclassification and under-reporting between MASLD and MetALD in the absence of more objective tests, such as phosphatidylethanol, which is a major limitation of this study [26]. Moreover, the population with MetALD was relatively small and underpowered for formal equivalence testing and should be interpreted with caution. Another limitation is the absence of liver biopsy, which was not feasible for all clinic patients, and the use of MRE as a reference, which, although accurate, is not a substitute for histopathology. Lastly, the selected laboratory variables had minimal missingness, and the primary endpoints remained unaffected.

In conclusion, in a tertiary liver clinic cohort with MRI-PDFF-confirmed steatosis and MRE as the reference for advanced fibrosis, we demonstrated that simple, widely implemented blood-based NITs, specifically FIB-4 and NFS, retain clinically useful accuracy in the newly defined MetALD subgroup. The NITs performed comparably to MASLD and support extension of guideline-concordant triage pathways to MetALD. APRI underperformed and should not be relied upon for fibrosis stratification in SLD. These data address an important evidence gap created by the new SLD nomenclature and offer pragmatic support for scalable risk stratification of MetALD in real-world specialty care.

## Data Availability

The data underlying this study are available from the corresponding author upon reasonable request.
